# Invasive Cardiac Lipoma: a case report and review of literature

**DOI:** 10.1186/s12872-016-0465-2

**Published:** 2017-01-14

**Authors:** Jason D’Souza, Rajesh Shah, Aamer Abbass, Jeremy R. Burt, Aditya Goud, Chanukya Dahagam

**Affiliations:** 1 Department of Internal Medicine, Florida Hospital, 2501 N. Orange Ave, Ste—235, Orlando, FL 32804 USA; 2 Department of Cardiology, Florida Hospital, 251 Maitland Ave #116, Altamonte Sp, FL 32701 USA; 3 Department of Radiology, Florida Hospital, 601 E. Rollins, Orlando, FL 32803 USA; 4 Department of Internal Medicine, MedStar Health, 9000 Franklin square drive, Baltimore, MD 21237 USA

**Keywords:** Case report, Cardiac lipoma, Benign cardiac tumor, Liposarcoma

## Abstract

**Background:**

Cardiac lipomas are rare benign tumors of the heart. They are usually asymptomatic and are thus most often diagnosed on autopsies. Symptoms, when present, depend upon the location within the heart. Typical locations are the endocardium of the right atrium and the left ventricle. Diagnostic modality of choice is cardiac MRI. Treatment guidelines have not yet been established due to the very low prevalence of these tumors and are thus guided by the patient’s symptomatology.

**Case presentation:**

We describe a case of an invasive cardiac lipoma, wherein the initial symptom of the patient was shortness of breath. Although the echocardiogram visualized the tumor in the right atrium, a cardiac MRI was performed for better tissue characterization. The MRI revealed a large, fat containing, septated mass in the right atrium with invasion into the inter-atrial septum and inferior cavoatrial junction. There was also invasion of the coronary sinus along the inferior and left lateral aspect of the posterior atrioventricular groove. Although the mass appeared to represent a lipoma by imaging characteristics, the unusual extension into the coronary sinus led to consideration of a low-grade liposarcoma in the differential. Thus a pre-operative biopsy was performed along with MDM2 gene amplification to rule out a liposarcoma preceding surgical excision.

**Conclusion:**

Cardiac lipomas are well-characterized on cardiac MRI, which is the diagnostic modality of choice. Typical locations are the right atrium and the left ventricle. However, in those with atypical features such as invasion of the coronary sinus, pre-operative biopsy for histopathologic confirmation is imperative to exclude well-differentiated liposarcoma. Our patient with a simple lipoma underwent partial resection to relieve symptoms. We discuss prognosis and treatment of symptomatic cardiac lipomas.

## Background

Primary tumors of the heart are uncommon. However, in the current era of sophisticated diagnostic imaging, their prevalence is increasing. The overall prevalence of primary cardiac tumors is between 0.17 and 0.19% [1]. Of these, 75% are benign, with cardiac lipomas representing only 8.4% of primary cardiac tumors [2]. Albeit benign, lipomas can be symptomatic depending on their location within the heart. They originate from the subendocardium (50%), subepicardium (25%) or myocardium (25%) and are of varying sizes. Typical locations include the right atrium and the left ventricle [3]. Distinction from lipomatous hypertrophy of the myocardium on imaging can be difficult. We report a unique case of a large cardiac lipoma, which although benign, appeared to have a malignant potential based on aggressive imaging features.

## Case presentation

**Fig. 1:**
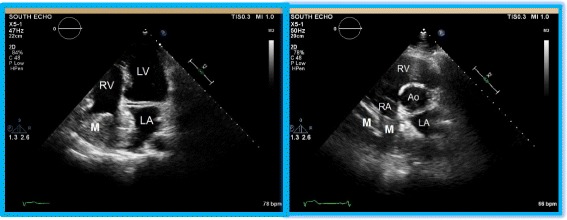
Echocardiographic imaging of the cardiac lipoma. The apical four chamber view and the parasternal short axis view clearly demonstrate the mass (M) originating from the free wall of the right atrium. The mass almost completely obliterating the right atrial cavity. Also notable is the involvement of the inter-atrial septum by this heterogenous mass. RA—right atrium; LA—left atrium; RV—right ventricle; LV—left ventricle; Ao—aorta

A 33 year old Caucasian gentleman presented to a local hospital with chronic right flank pain and was being evaluated for multiple cystic masses in the liver. On review of symptoms, he mentioned that he occasionally experienced shortness of breath, more so with exertion. His vitals, physical examination and laboratory data were unremarkable. Electrocardiogram displayed a normal sinus rhythm. However, his symptoms prompted an echocardiogram, which revealed a large, heterogeneous, non-mobile, obstructive mass in the right atrium that measured 45 x 40 mm and appeared to involve the inter-atrial septum as well, as shown in Fig. 1. Given these findings, cardiac MRI was performed for better tissue characterization.

**Fig. 2:**
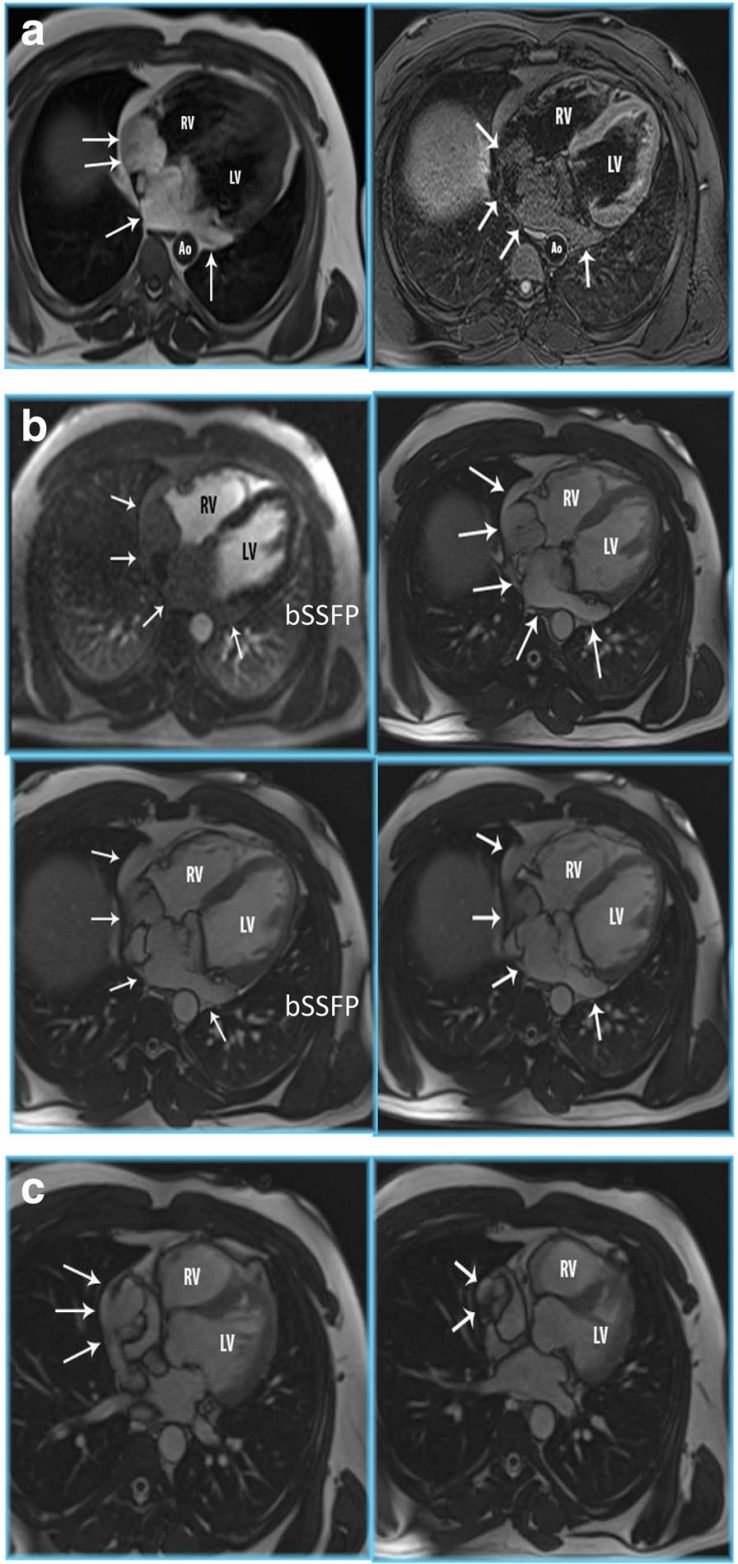
Cardiac MRI of the cardiac lipoma. **a** Cardiac MR double (DIR) and triple inversion recovery (TIR) sequences clearly demonstrates a large fat containing mass in right atrium, interatrial septum and coronary sinus (*white arrows*). (RV = right ventricle; LV = left ventricle; Ao = descending aorta). **b** Axial T1 postcontrast (T1 w) and balanced steady state free precession (bSSFP) cardiac MR demonstrates a large fat containing mass in the right atrium, interatrial septum and coronary sinus (*white arrows*). No enhancing components suggests this is a simple lipoma (RV = right ventricle; LV = left ventricle). **c** Balanced steady state free precession CMR images in HLA shows the lipoma extending from the right atrium into the IVC (*white arrows*). (RV = right ventricle; LV = left ventricle)

The MRI demonstrated a lesion with increased T1 signal involving the myocardium of the right atrium and the inter-atrial septum. Multiple thin septations were noted in the mass. The mass had slightly decreased T2 signal relative to myocardium. The tumor also extended into the inferior vena cava and coronary sinus. The right atrial component of the mass measured 39 x 38 mm, while the component involving the inter-atrial septum and extending into the coronary sinus measured 93 x 47 mm with a cranio-caudal dimension of 65 mm. A venacavogram was also performed, which confirmed the presence of the intracardiac mass in the right atrium that extended caudally into the central intrahepatic segment of the inferior vena cava. The MRI findings are as displayed in Fig. 2 (a-c).

**Fig. 3:**
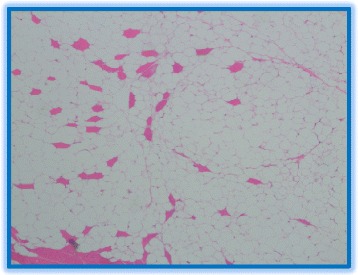
Histopathology of the tumor. Histopathology of the lipoma specimen reveals mature adipocytes and absence of other soft tissue components which otherwise would have indicated a liposarcoma

Although the imaging findings were suggestive of an intracardiac lipoma, given the septations, size and invasion into the inferior cavoatrial junction and coronary sinus, a well-differentiated liposarcoma could not be excluded. Therefore, a pre-operative diagnosis was imperative before considering major cardiothoracic surgery with its associated morbidity and mortality. The right atrial component of the mass was biopsied by a right heart catheterization with the guidance of a transesophageal echocardiogram. Histopathology confirmed a lipoma (Fig. 3) with a negative MDM2 gene amplification by FISH, thus ruling out liposarcoma.

After weighing the risks and benefits of surgical resection versus conservative management with close follow-up, the patient chose to proceed with surgery. The patient underwent complete resection of the right atrial mass and debulking of the inter-atrial septal component of the tumor. A complete resection of the inter-atrial septum was not performed as this would increase the complexity of the surgery and the risks involved. Intra-operatively, it was also noted that the tumor could be palpated extending for about 2–3 cm into the coronary sinus. After extensive discussion with other cardiothoracic specialists and considering the potential risks and benefits, a decision was made to not excise the remaining components of the tumor. Ultimately, the remaining components of the tumor were felt to be unrelated to the patient’s symptomatology. The patient tolerated the procedure well without any complications and had an uneventful recovery. Three months into his follow-up, the patient feels well and has no shortness of breath.

## Discussion

Cardiac lipomas are benign tumors and account for a small number of the primary tumors of the heart [2]. They can occur in any age group, but are most prevalent between ages 40–60 years [3]. They are usually asymptomatic and hence remain undetected or are discovered incidentally. Symptoms, when present, are varied and depend on the location of the heart involved. Tumors in the subepicardial region can create a mass effect on nearby structures. They can cause angina if they compress the coronary arteries or they can reduce systolic function by compressing on the left ventricle. Tumors in the myocardium can infiltrate the electrical circuit and be a nidus for arrhythmogenesis. That being said, the most common location is the subendocardial region, with a particular predilection for the right atrium and the left ventricle. Depending on the chamber involved and the size of the mass, they can cause obstruction of flow and congestive heart failure [4, 5]. Embolization is a rare phenomenon because lipomas are typically encapsulated.

Diagnostic evaluation begins with an echocardiogram that offers a simple and non-invasive approach. However, it may not be able to visualize smaller tumors. Furthermore, echocardiography often cannot conclusively differentiate between lipomas and other primary tumors of the heart [6]. In such situations, computerized axial tomography and magnetic resonance imaging may be of additional value. While lipomas appear to not enhance and have homogenous high signal intensity on T1 and T2 weighted images, liposarcomas typically contain solid, enhancing components intermixed with areas of fat signal [1]. Another differential diagnosis for a fat containing mass within the heart is lipomatous hypertrophy of the interatrial septum. In reality, lipomatous hypertrophy is a misnomer as the pathophysiology involves hyperplasia of adipocytes as opposed to hypertrophy. Unlike lipomas, these are unencapsulated, spare the fossa ovalis and thus bear a dumbbell shape [6]. In our case, although the MRI findings were nearly conclusive for lipoma based on tissue characteristics, the multiple septations, extensive involvement of the right atrium and interatrial septum, as well as the inferior cavoatrial junction and coronary sinus raised concerns for well-differentiated liposarcoma. Thus, a pre-operative biopsy was performed which confirmed a simple lipoma with no evidence for malignancy. The sample was negative for MDM2 gene amplification which has a 93.5% sensitivity for detecting atypical and malignant lipomatous tumors [7]. To complete the work-up of a cardiac tumor, a left heart catheterization becomes important to delineate the coronary anatomy, particularly for those who would eventually need surgery. Apart from evaluating for coronary artery disease, the benefit of a catheterization is two-fold. Not only does it provide information on whether the tumor is compromising vascular supply to the myocardium, but it also gives details on the vascular supply to the tumor [1].

Given their low prevalence, there are no randomized clinical trials to guide treatment. A surgical approach is justified in patients who are symptomatic, to alleviate symptoms and to prevent progression of disease. However, treatment in asymptomatic patients poses a serious dilemma to the patient and the clinician as there is no consensus to date [4].

## Conclusion

Cardiac lipomas are rare benign tumors of the heart. They are well-characterized on cardiac MRIs. However, in those with atypical features, pre-operative biopsy and confirmation is imperative in assessing prognosis and guiding management.

## Abbreviations

FISH: Fluorescence in situ hybridization
IVC: Inferior vena cava
MDM2: Mouse double minute 2 homolog
MRI: Magnetic resonance imaging
